# Cognitive Orientation to daily Occupational Performance (CO-OP) for mood, anxiety, and adjustment disorders: a pilot study

**DOI:** 10.3389/fpsyt.2024.1428811

**Published:** 2024-09-26

**Authors:** Su Ren Wong, Mu Rong Chan, Edlina Chong, Karina Michelle Dancza

**Affiliations:** ^1^ Department of Rehabilitation, National University Hospital, Singapore, Singapore; ^2^ Health and Social Sciences, Singapore Institute of Technology, Singapore, Singapore; ^3^ Occupational Therapy, Tan Tock Seng Hospital, Singapore, Singapore; ^4^ Ambulatory Services, Kwong Wai Shiu Hospital, Singapore, Singapore

**Keywords:** occupational therapy, mental health, Cognitive Orientation to daily Occupational Performance, CO-OP, mood disorder, anxiety disorder, adjustment disorder

## Abstract

**Introduction:**

Global mental health issues, particularly anxiety and depression, significantly impact people’s everyday activities. While psychotherapies are commonly used, there is a growing interest in problem-solving approaches within mental health. These approaches focus on enabling individuals to develop personalized strategies to address emotional and psychological challenges and enhance their engagement in meaningful activities, known as occupational performance. This paper examines the feasibility of the Cognitive Orientation to daily Occupational Performance (CO-OP) in assisting adults with mood, anxiety, or adjustment disorders.

**Method:**

The study employed a mixed methods single-subject design with replication, using an inductive/deductive approach for qualitative analysis. Ethical approval was obtained, and participants were recruited from a Singaporean hospital’s occupational therapy service. CO-OP sessions were conducted either in-person or via telehealth. The intervention involved setting goals collaboratively, followed by weekly sessions over 10 weeks. Various data sources, including demographics, field notes, recordings of sessions, assessments and interviews were collected. Data analysis involved comparing pre- and post-intervention scores, thematic analysis of interviews, and triangulation of quantitative and qualitative data for validity. The study results are organized according to five feasibility domains: acceptability, demand, implementation, practicality, and limited efficacy.

**Results:**

A total of 10 participants, mostly female, were recruited, with two dropping out during the baseline phase. All remaining participants completed the intervention and 1 month follow-up data collection. CO-OP was perceived as acceptable and beneficial in enhancing occupational performance, satisfaction and managing mood and anxiety symptoms. Participants expressed increased confidence and self-efficacy but desired continued therapist support for strategy application and reinforcement.

**Discussion:**

Participants generally embraced CO-OP, favoring its personalized nature over therapist-directed approaches, with high retention rates observed. Building a strong therapeutic relationship was essential. Also using complementary approaches like supportive counseling proved beneficial. CO-OP emerges as a viable intervention alongside existing therapy approaches, offering a promising avenue for addressing the complex needs of individuals with mental health conditions.

## 1 Introduction

Mental health concerns, particularly anxiety and depression, are increasing globally (1). They remain major causes of disability worldwide (2) and significantly impact various aspects of peoples’ lives (3, 4). These disorders can hinder everyday activities such as self-care, work, home management, and social interactions (5, 6). Occupational therapists in mental health settings play a crucial role in supporting people to cope with emotional and psychological challenges and facilitate their engagement in meaningful activities, known as *occupational performance*.

Psychotherapies are commonly used to support individuals experiencing anxiety and depression (7). These therapies encompass various mental health approaches aimed at improving psychological, emotional, and behavioral symptoms. While psychotherapies are widely researched and found to be effective in reducing symptoms, their impact on improving occupational performance remains inconclusive (8–11). Although occupational therapists in mental health settings have traditionally applied such approaches, there is a growing interest within occupational therapy in problem-solving interventions directly addressing individuals’ ability to perform their occupations, referred to as occupation-centered practice (62).

One such problem-solving approach that focuses on enabling individuals to develop personalized strategies for achieving their occupational goals is the Cognitive Orientation to daily Occupational Performance (CO-OP) (12). CO-OP is a metacognitive, person-centered approach that empowers individuals to identify cognitive strategies for enhancing occupational performance through an iterative process of performance analysis and guided discovery (13). The objective of CO-OP is to improve occupational performance through skill acquisition, utilization of cognitive strategies, application of learning to real-world activities, and transfer of learning to novel situations. The intervention format is based on the use of a global problem-solving strategy, “Goal-Plan-Do-Check” (GPDC) (12, p. 48). CO-OP is a complex intervention that consists of seven main elements: namely: 1) Client-chosen goals, 2) Dynamic performance analysis, 3) Cognitive strategy use 4) Guided Discovery, 5) Enabling Principles, 6) Significant other involvement and 7) Intervention format (12, p. 48).

CO-OP, originally designed for children with developmental coordination disorder (14, 15), has been further researched with a range of other pediatric populations, such as children with neurodevelopmental disorders (16), acquired brain injury (17), intellectual disability, and cerebral palsy (18, 19). Within the adult population, CO-OP has been found to be effective in patients with motor and cognitive difficulties post-stroke and traumatic brain injury (20, 21). Additionally, CO-OP has been explored in adult populations with hand injury, Parkinson’s disease, and various conditions associated with cognitive impairment (22–25).

The evidence for using CO-OP with adult populations is evolving and indicates positive outcomes. For instance, the global problem-strategy taught in CO-OP was found to be effective in promoting long-term gains in occupational performance and satisfaction with goals set by individuals following traumatic brain injury (26). Results also demonstrated that the effects of global metacognitive training strategies were generalizable to different contexts and transferable to new goals. In a randomized controlled trial of CO-OP with upper extremity burns, patients showed improvements in occupational performance and satisfaction, in addition to improvements in anxiety and depressive symptoms (27). However, while conducting CO-OP with adult populations, some adaptations to the original CO-OP protocol have been reported as necessary, such as modifying the involvement of significant others or adjusting the duration and frequency of treatment sessions (21, 26, 28, 29).

Given the initial success of CO-OP with diverse adult populations experiencing mood and cognitive difficulties (27), there is potential for CO-OP’s guided, problem-solving approach to be beneficial for people with other mental health conditions. This paper will address the feasibility of administering CO-OP to individuals with mental health issues. Feasibility will be assessed based on five relevant domains described by Bowen et al. (30), namely acceptability, demand, implementation, practicality, and limited efficacy.

## 2 Materials and methods

### 2.1 Study design

A mixed methods single-subject design with replication was used for the study, employing an inductive/deductive approach for qualitative analysis (31, 32). This study received approval from the Domain Specific Review Board (No. 2019/00049) of the National Healthcare Group and the Singapore Institute of Technology Institutional Review Board (No. 2019121). Written informed consent was obtained. Participants paid for their sessions; however, they were compensated with a $30 supermarket voucher per visit.

### 2.2 Participants

Participants were purposively recruited from the occupational therapy service at a large tertiary hospital in Singapore between 2019 and 2021. This service was seeing a large group of people with mood, anxiety and adjustment disorders and were interested in possible interventions for this population. Thus, the inclusion criteria were: (1) a diagnosis of a mood, anxiety, or adjustment disorder; (2) aged between 21 and 65 years; (3) medically stable; (4) proficient in English; (5) capable of providing informed consent; and (6) referred for outpatient mental health occupational therapy services. Exclusion criteria were people with a degenerative neurological condition or people who were unable to identify goals they wished to address during the sessions.

### 2.3 Procedures

CO-OP sessions were conducted in an outpatient hospital setting. Due to the evolving COVID-19 situation at this time, participants recruited in 2021 were given the option to choose between in-person or telehealth sessions. Despite this provision, only one person opted for telehealth for two out of their ten sessions. The intervention was delivered by an experienced occupational therapist certified in the CO-OP approach. Hour-long individual sessions took place once weekly, spanning over 10 weeks. The first session involved collaborative goal setting, facilitated by the therapist. Subsequent sessions involved the therapist introducing the global problem-solving approach (GPDC), reviewing goal attainment, and refining strategies generated and applied by participants if goals were not met. Additionally, the therapist provided session handouts that summarized the strategies and plans for each participant to take home after each session.

### 2.4 Data collection

**Table 1** provides a summary of the quantitative and qualitative data sources collected and triangulated to assess the five domains of feasibility. The study gathered qualitative data via semi-structured interviews, field notes, and video/audio recordings across all five criteria (Acceptability, Demand, Implementation, Practicality, and Limited Efficacy). The study employed several quantitative measures to complement the qualitative insights. For assessing Acceptability, the Treatment Acceptability/Adherence Scale (TAAS) provided numerical data on how acceptable participants found the intervention. Demand was assessed via the recruitment and retention rates. To ensure proper Implementation, the CO-OP Fidelity Checklist offered quantitative data on the fidelity of the intervention’s execution according to established standards. Additionally, the study utilized several other quantitative measures for determining Limited Efficacy: the Canadian Occupational Performance Measure (COPM) and the Performance Quality Rating Scale (PQRS) provided scores reflecting the quality of performance in various activities from both the participant’s and therapist’s perspective respectively; the Patient Health Questionnaire-9 (PHQ-9) measured the severity of depressive symptoms; and the Generalized Anxiety Disorder-7 scale (GAD-7) quantified the severity of anxiety symptoms.

**Table 1:** Data sources and the five domains of feasibility.

	Acceptability	Demand	Implementation	Practicality	Limited Efficacy
Demographics questionnaire					
Field notes^1^	x	x	x	x	x
Video/audio recordings of sessions^2^	x	x	x	x	x
Semi-structured interviews^3^	x	x	x	x	x
Recruitment and retention^4^		x			
CO-OP fidelity checklist^5^			x		
Canadian Occupational Performance Measure (COPM)^6^					x
Performance Quality Rating Scale (PQRS)^7^					x
Patient Health Questionnaire-9 (PHQ-9)^8^					x
Generalized Anxiety Disorder-7 scale (GAD-7)^9^					x
Treatment Acceptability/Adherence Scale (TAAS)^10^	x				

1 The therapist wrote field notes after each session reflecting on implementation of CO-OP, possible adaptations, and feedback (60).

2 CO-OP sessions were audio and video recorded with permission from the participant to allow for post session analysis and interpretation of behavior (68).

3 Conducted to understand participants’ subjective experience of the intervention, providing insight into participant’s perspective of feasibility (59). The interviews were video, and audio recorded and ranged from 30 to 60 minutes long.

4 Descriptive data were collated regarding recruitment rate, retention rate, number of sessions rescheduled, and reasons for dropping out.

5 Examines therapist’s adherence to the CO-OP protocol for clinical or research purposes (61, p.16). The fidelity checklist was independently rated using the video of the sessions by at least two researcher assistants. In addition, a blinded CO-OP trained therapist randomly selected and scored 20% of the rated CO-OP sessions. 100% similarity was achieved.

6 COPM is a standardized semi-structured interview to elicit goals for intervention sessions (65). It assesses self-perceived performance and satisfaction of identified occupational performance goals on a 10-point scale. Improvements by 2 or more points on pre-post scores of COPM indicate clinically significant improvements (65). COPM has been demonstrated to be an appropriate measure in detecting changes in levels of performance and satisfaction in persons with mental health disorders (59).

7 PQRS is a therapist-rated observational measure of participant’s performance quality in meaningful, personal daily activities. PQRS was designed to complement COPM by capturing therapists' perception of goal performance (66). Performance is rated on a 10-point scale, where ‘1’ indicates that the activity was not performed at all, while ‘10’ indicates that the activity was performed very well.

8 PHQ-9 is a 9-item self-reported screening tool for severity of depressive symptoms (63). Higher scores indicate higher severity of symptoms. A change of five points or more suggests clinical significance. The PHQ-9 reported excellent internal and test-retest reliability, and good construct validity (64).

9 GAD-7 is a 7-item self-reported measure designed to screen and assess the severity of symptoms of anxiety (69). A score of 10 and above suggests the presence of anxiety. The scale demonstrated good test-retest reliability and is specific and sensitive in measuring symptom severity (64).

10 TAAS is a self-report measure consisting of 10 statements describing participant’s response to treatment. Items are scored on a seven-point Likert scale (strongly disagree, neutral, strongly disagree), with a total score ranging between 10 and 70. A higher score suggests greater acceptability and predicted adherence to treatment. TAAS has been used to study novel treatment approaches for anxiety and related problems. It can also aid in providing information to further improve the novel approach based on the participant’s self-reported answers (67).

As shown in **Table 2**, data was collected at baseline (T1), CO-OP sessions (T2), final CO-OP session (T3) and 1-month follow-up (T4). At T1, the baseline measures were repeated weekly for three weeks. The CO-OP fidelity checklist (70) was rated based on sessions three, six and nine for each participant as per Yosef et al. (33). For participants who did not complete all ten weekly sessions, one session from each third of the total number of sessions were sampled. All measures were administered by unblinded research assistants, except for the COPM and PQRS, which were completed by the occupational therapist.

**Table 2:** Timeline of sources of data collection.

	T1 Baseline	T2 CO-OP sessions (biweekly)	T3 Final CO-OP session	T4 1 month follow up
Demographics questionnaire	x			
Field notes		x	x	
Video/audio recordings of sessions		x	x	
Semi-structured interviews			x	x
Recruitment and retention	x	x		
CO-OP fidelity checklist		x		
Canadian Occupational Performance Measure (COPM)	x		x	
Performance Quality Rating Scale (PQRS)	x		x	
Patient Health Questionnaire-9 (PHQ-9)	x	x	x	x
Generalized Anxiety Disorder-7 scale (GAD-7)	x	x	x	x
Treatment Acceptability/Adherence Scale (TAAS)		x	x	

### 2.5 Data analysis and reliability

To examine primary efficacy in improving occupational performance, pre- and post- intervention scores were compared for COPM and PQRS. COPM data was analyzed for normality using the Shapiro-Wilk test. The Wilcoxon Signed Rank test was used as not all the data was normally distributed. In addition, to examine efficacy in symptom reduction, scores for PHQ-9 and GAD-7 were plotted on graphs for visual analysis of levels, trends and consistency (34). To improve validity of findings, quantitative data was triangulated with qualitative interviews (35).

Interviews were de-identified and transcribed verbatim. An inductive/deductive thematic analysis approach was used to allow targeted analysis of study objectives while identifying new patterns in data (28, 32, 36). A coding frame was developed following Roberts et al. (32), with reference to the format by Boyatzis (37). This coding frame ensured greater inter-rater reliability for the coding of qualitative data (32). The coding frame included predetermined categories based on the five domains of feasibility (30), and a literature review of CO-OP. The frame was iteratively refined after its application to two initial transcripts by two study team members through discussion and consensus. Using NVivo 12 software, the frame was applied to the remaining transcripts, with codes sorted into predetermined categories, while new codes were derived inductively.

## 3 Results

### 3.1 Demographics and description of participants

A total of 10 participants were recruited (referred to as P1 to P10), among whom nine were female and one was male (P10). Two participants dropped out during the baseline phase; P6 cited a change in priorities, while P10 was uncontactable. The demographic information of the remaining eight participants is presented in **Table 3**. All the participants met the inclusion criteria in having mental health diagnoses; in addition, three participants had co-morbid diagnoses (i.e. Autism Spectrum Disorder, Attention Deficit-Hyperactivity, Learning Difficulties, and Social Communication Disorder). All eight participants completed the CO-OP sessions and follow-up phases. The results of the study are organized according to the five domains of feasibility chosen, namely acceptability, demand, implementation, practicality, and limited efficacy (30).

**Table 3:** Participants demographic information.

	Age	Sex	Race	Diagnosis	Employment status (Profession)	Marital status
P1	34	Female	Chinese	Anxiety with history of Autism Spectrum Disorder, Attention Deficit-Hyperactivity Disorder with learning difficulties	Unemployed (Library training during study)	Single
P2	41	Female	Malay	Generalized Anxiety Disorder	Unemployed	Married
P3	22	Female	Chinese	Adjustment reaction/disorder	Student	Single
P4	57	Female	Chinese	Mixed Anxiety Depression	Unemployed	Married (Divorce during study)
P5	29	Female	Malay	Anxiety Depression	Employed (Financial consultant)	Single (Married during study)
P7	24	Female	Chinese	Autism Spectrum Disorder with depression and mood dysregulation	Unemployed (Employed as retail assistant during study)	Single
P8	21	Female	Chinese	Social Communication Disorder – Adjustment Disorder	Student	Single
P9	45	Female	Javanese	Adjustment disorder with depressed mood and anxiety	Unemployed	Married

### 3.2 Acceptability

#### 3.2.1 Overall acceptability of the approach

Mean TAAS scores consistently trended in the upper range, ranging between 68.6% to 92.4%, with minimal fluctuations. This indicates moderate to high acceptability, which was consistent with participants expressing generally positive views towards the CO-OP approach.

Participants expressed enjoyment and increased motivation to participate in CO-OP sessions compared to other therapies, as highlighted by P8:

*“I find that it was a good experience, and that I would definitely like to go through this therapy again, if possible, to better address the goals that I’ve set for myself”*.

A common factor mentioned was how previously experienced therapies were more prescriptive, whereas CO-OP was individualized and personally meaningful. For example, P5 stated:

*“I told her [the CO-OP therapist] that she’s not the first person I’ve seen. I always end up feeling like whatever the person teaches or recommends [to] me doesn’t work eventually, so I give up and don’t want to meet them anymore. But for her, it’s collaborative”.*

During the T4 interview, all participants expressed that they would recommend CO-OP to individuals facing similar difficulties.

#### 3.2.2 Perceived appropriateness of CO-OP key elements

Participants expressed how they valued the active partnership fostered throughout the CO-OP sessions, contrasting it with previously experienced therapist-directed approaches. They perceived this partnership approach as enabling greater individualization of goals and plans, thus making them more realistic and personally meaningful. For example, P1 expressed preference for the CO-OP approach, stating,

*“I prefer [a partnership approach] because then they’ll be [considering] what you like and what is suited for you, and they won’t throw me into something that I probably cannot actually do. And then they’ll discuss different ways of handling [the situation/problem], instead of you know, just telling me what to do”.*

Despite the value participants placed on working in partnership with the therapist on their goals, the use of the guided discovery questioning technique was occasionally frustrating, particularly one participant who found it demanding in terms of their active engagement. However, the approach was generally positively received and reportedly facilitated feelings of autonomy, the development of personally meaningful plans, and promoted follow-through of the self-determined strategies.

Additionally, the Goal-Plan-Do-Check (GPDC) strategy was perceived as valuable for developing effective strategies, fostering a willingness for trial and error, and organizing thoughts. However, two participants reported not using it outside of the CO-OP sessions due to a lack of new situations to do so.

### 3.3 Demand

All participants who started the CO-OP sessions were able to complete the study, (i.e. data collection at all time points), resulting in a retention rate of 100%. However, only half of the participants (n=4) completed the full protocol of 10 weekly sessions. The 10 weekly sessions were not necessary for P2, who achieved all her goals by session seven. Participants P4 and P7 missed one weekly session, while participant P5 missed two weekly sessions with the main reasons being feeling unwell or forgetting appointments. Rescheduling was attempted and participants indicated their desire to continue, however the full 10 weekly sessions were not possible within the timeframe of the study. All participants completed the final data collection at T3 (final session) and T4 (one month follow-up).

Among the participants given the option for telehealth or in-person sessions, only P8 opted for telehealth for 2 out of 10 CO-OP sessions. They shared that collaboration was more difficult via telehealth, although it was more convenient than face-to-face sessions. In addition, during the post-intervention interview, P7 expressed a preference for in-person sessions due to the need for *“human touch”*. P9 elaborated further and expressed,

*“Sometimes, the reception is not good, I cannot hear properly, I cannot see you all properly, and then I will get angry”*.

### 3.4 Implementation

#### 3.4.1 Success or failure and degree of execution

The CO-OP fidelity checklist scores were used to rate sessions 2, 4 and 6 for P2 and P5 who completed seven sessions, and sessions 3, 6 and 8 were rated for P2 and P7 who completed nine sessions. Fidelity for P1 was not rated, as consent was not given for recordings.

Overall ‘across session’ fidelity of 100% was obtained, with mean ‘within session’ fidelity of 96.8% for all participants, indicating that the therapist had a high quality of execution and compliance to the CO-OP protocol. For all sessions, above 80% of items had high quality of execution except one session for P3 and P7, where a large portion of the session was focused on supportive counselling instead of engaging in CO-OP.

#### 3.4.2 Factors affecting implementation

Participants took time to clarify their desired goals, leading to frequent changes. As the therapist noted,

*“One challenge is the constant change in goals and the need to remain relevant to new situations. Goals often become irrelevant or delayed due to changes in mood or life situations”.*

In addition, administering the Performance Quality Rating Scale (PQRS) and Dynamic Performance Analysis posed challenges due to the nature of goals selected by participants. Many goals related to social interactions or changes to routines could not be directly observed during sessions and had to be evaluated through detailed discussions.

Participants’ limited knowledge of what was involved in making progress towards their goals also made using the guided discovery technique challenging. The therapist found facilitating guided discovery *“taxing”*, requiring a delicate balance between providing task knowledge and encouraging self-discovery. Sometimes, the therapist directly supplemented participants’ knowledge, particularly when extensive research would be required or when time constraints made self-discovery impractical. The therapist explained,

*“It seemed difficult for her to generate ideas, and given her circumstances and lack of exposure to potential strategies, exploring on her own would have been challenging. Therefore, I decided to directly supplement task knowledge”.*

The therapist’s skilled management of participants’ low mood and interpersonal events facilitated the implementation process. The therapist employed various strategies, such as supportive counseling and validating emotions, guided by other therapeutic approaches. Recognizing and switching approaches as needed enabled participants to experience catharsis and subsequently increased their engagement in the CO-OP sessions. For example, one participant mentioned to the therapist that,

*“Just by talking about my situation in the last session, I felt much better. It almost felt like the last session prepared us to work more effectively today on problem-solving. I was able to dive into it and go straight into CO-OP today”.*

### 3.5 Practicality

Participants’ ability to learn, generalize, and transfer Goal-Plan-Do-Check (GPDC) and domain-specific strategies were examined. Some participants encountered difficulties recalling GPDC steps when asked, yet demonstrated implicit application. For example, five participants successfully transferred GPDC to other situations. One participant introduced GPDC to her project group, while another went beyond the original planned strategy and developed a new way of managing a crowded supermarket. P9 described,

*“I know the timing is like evening, people after office hours, they start to go [to the supermarket] already. So I go at noon, [or] about 2[pm]. Not a lot of people.”*

Another example was a participant who found the GPDC strategy application natural, applying it to manage her children’s behavior.

Participants expressed a lack of confidence at times in their plans. One participant voiced uncertainty regarding the effectiveness of her plans and sought assistance from the therapist to refine her ideas,

*“I can probably come up with some ideas but not every single idea so I might need help with, you know, fixing that to make sure it actually works.*” (P1)

Participants also expressed that their lack of confidence in their plans arose from apprehensions about unforeseen variables affecting plan success. For example, P8 reflected,

*“everyone’s responses might be different with regards to your plan,…[where] the other individual involved will be an unknown variable, so it’ll be difficult to go through the entire plan sometimes”*.

Some participants recognized the need for time to integrate strategies into their routines and develop new habits. P5 shared that,

*“I believe habit takes time … there’s a bit of a … challenge to implement that, because naturally, I see it a certain way … [and that cultivating new habits was] very uncomfortable … very time consuming”.*

Some participants expressed the need for continued therapist support beyond the completion of their goals due to persistent struggles managing their symptoms. P4 expressed concerns that she would regress in her improvements and struggle to push herself. She felt that she required external pressure from the therapist, to still be

*“accountable to this person, to tell this person that I’m moving on. It makes me stay on track, or else I will move backwards”*.

P5 also shared that she still experienced herself spiraling into negative emotions, and that

*“sometimes when the spiral is too deep it’s very difficult to catch yourself”.*

### 3.6 Limited efficacy

#### 3.6.1 Occupational performance

Participants and the therapist rated improvements in 83.3% and 91.7% of their occupational performance goals, respectively (**Table 4**). In the Canadian Occupational Performance Measure (COPM), all participants reported clinically significant improvement in performance of at least two goals, with half of the participants reporting clinically significant improvements in performance for all three goals. A Wilcoxon Signed Ranks test showed that there were statistically significant improvements in COPM performance scores from pre-CO-OP to post-CO-OP sessions (Z=36.00, p = .012).

**Table 4:** Participant goals and respective COPM and PQRS scores.

	Goals	PQRS		COPM Performance		*W* ^a^	COPM Satisfaction		*W* ^b^
				Mean (*SD*)^a^			Mean (*SD*)^a^		
		Start	End	Start	End		Start	End	
				4.33^b^	7.33^b^	36.00*	3.50^b^	7.67^b^	36.00*
P1	Find a job	3	8	4.33 *(0.58)*	6.67^c^ *(0.58)*		4.33 *(1.53)*	7.33^c^ *(1.15)*	
	Travel independently	2	9						
	To be able to use a public toilet	3	7						
P2	Eat regularly and healthily	7	7	4.33 *(1.53)*	7.33^c^ *(0.58)*		4.00 *(2.00)*	7.33^c^ *(0)*	
	Regular relaxation routine	3	8						
	Regular physical activities	2	7						
P3	Learn to play cajon	6	8	4.67 *(1.53)*	6.67^c^ *(2.89)*		3.33 *(3.46)*	6.33^c^ *(4.04)*	
	Engage in art once per week	6	10						
	Pre-sleep routine 3 times per week	6	9						
P4	Engage in a pre-sleep routine	1	7	4.33 *(3.06)*	7.33^c^ *(0.58)*		5.33 *(4.51)*	8.33^c^ *(1.53)*	
	Routinely take care of self	4	6						
	Prepare a meal 2-3 times a week	5	9						
P5	Shower every morning	7	10	4.67 *(3.21)*	7.33^c^ *(2.52)*		2.33 *(2.31)*	9.33^c^ *(1.15)*	
	Sleep from 1-7am	2	7						
	Go to office on Monday	4	5						
P7	Communicate in a less angry way	5	7	3.33 *(1.15)*	7.67^c^ *(0.58)*		5.33 *(1.53)*	8.00^c^ (1.00)	
	Style own hair	4	6						
	Manage emotions	3	7						
P8	Completing school tasks	5	7	3.33 *(1.53)*	7.67^c^ *(2.08)*		1.00 *(0)*	7.00^c^ *(2.00)*	
	Starting a conversation	4	7						
	Continuing a conversational topic	4	10						
P9	Manage feelings of numbness	2	8	1.00 *(0)*	7.00^c^ *(0)*		1.00 *(0)*	8.00^c^ *(0)*	
	Manage feeling overwhelmed by crowds	2	8						
	Communication with mother: assertive	3	8						

^a^ SD = standard deviation.^b^ W = Wilcoxon Signed Rank test. As Wilcoxon Signed Rank Test was used to analysis the difference in COPM Performance and Satisfaction scores, median scores are presented.^c^ For COPM, a 2-point change indicates a clinically significant difference.

*p > 0.05.

The positive outcomes observed were consistent with participants’ personal experiences shared in the interviews, where they commonly perceived CO-OP as a beneficial intervention. They expressed satisfaction with their enhanced goal performance and their newfound ability to effectively manage their condition and associated challenges. For example, P9 articulated their contentment, stating,

*“I’m satisfied right now lah like, I got improvement.”*

Similarly, P2 recounted moments of breathlessness during public transport journeys but highlighted the efficacy of CO-OP strategies, noting,

*“Eh! Okay what, I can manage myself.”*

Interestingly, P5 acknowledged minimal changes in mood and anxiety symptoms but still perceived growth, stating,

*“there’s growth because, I can sleep - even though [it is] not to the benchmark I want. And I can [also] go [to the] office [which were my goals].”*

These transformations were evident not only to the participants but also to those within their social circles. P5 spoke about how their friends noted the positive changes, observing,

*“When I’m having problems, I literally don’t meet anyone … now, I’m starting to have activities and they [my friends] see improvements.”*

Likewise, P9 shared how her husband’s friends remarked,

*“Your wife looks so different now”*

after she commenced the CO-OP sessions.

The problem-solving focus in CO-OP was perceived as a pivotal catalyst for change. P4 detailed its impact on her progress, stating,

*“[The CO-OP approach] changed the way I look at things.”*

Moreover, participants reported heightened confidence to engage in activities they previously avoided. P3 expressed this sentiment, saying,

*“My confidence level [to do activities] has actually increased by 100%.”*

#### 3.6.2 Mood and anxiety symptoms

**Figure 1:**
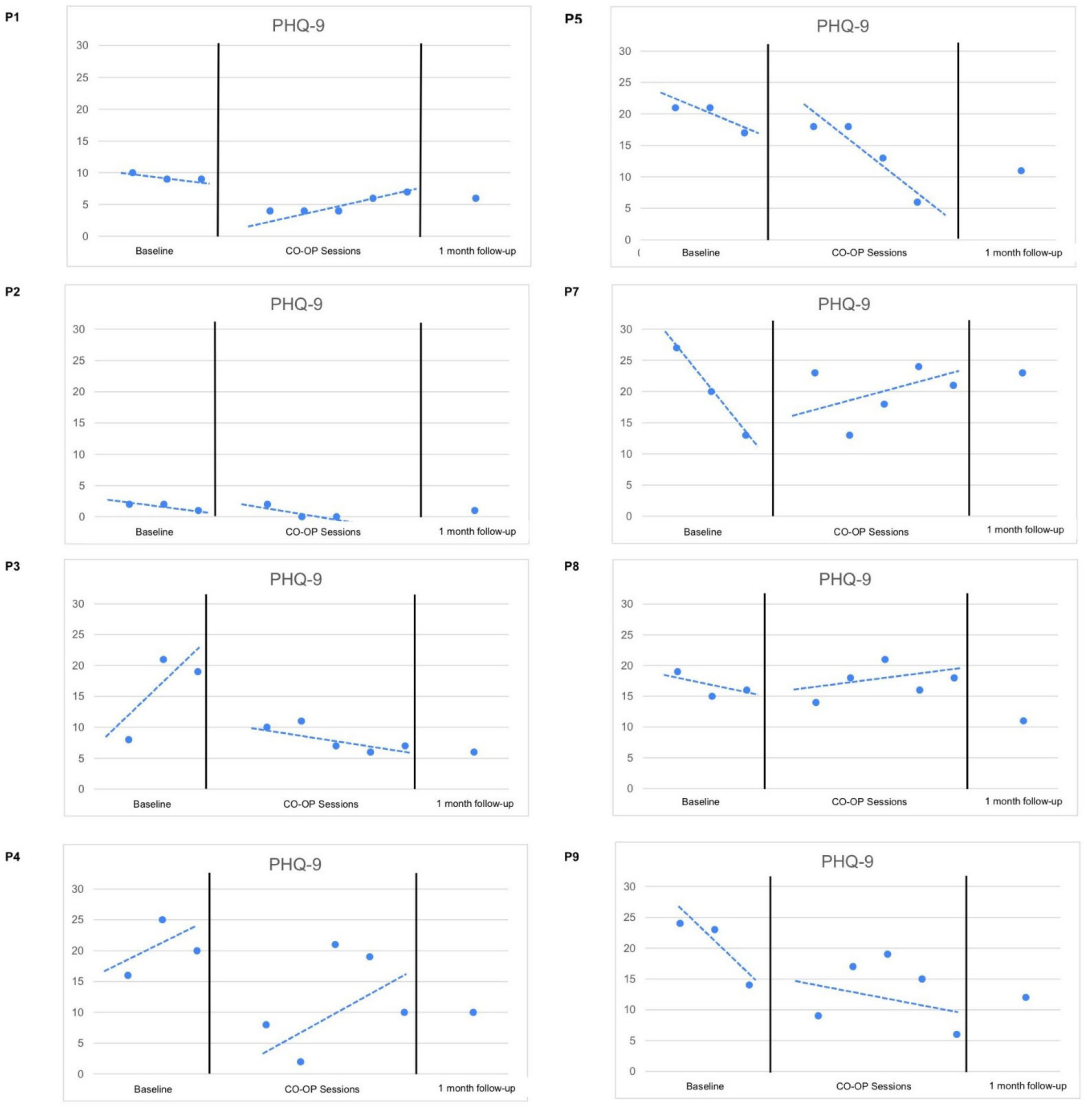
PHQ-9 Trends.

**Figure 2:**
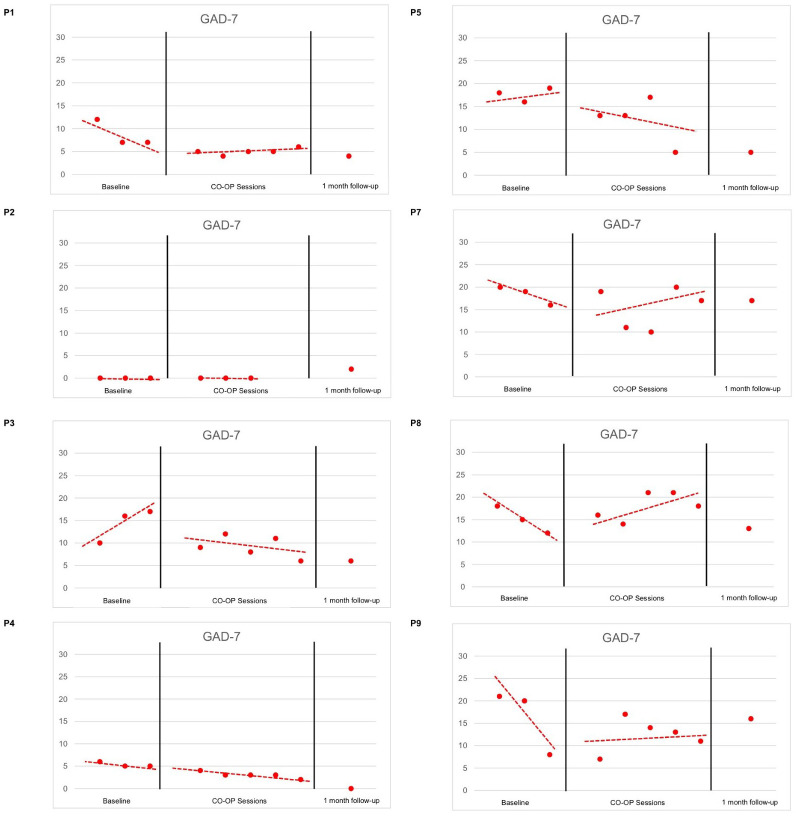
GAD-7 Trends.

**Figures 1** and **2** display the changes in depressive and anxiety symptom severity from T1 (baseline) to T2 (CO-OP sessions), T3 (final CO-OP session), and T4 (1-month follow-up).

##### 3.6.2.1 Trends

When examining the depressive and anxiety symptoms, P1, P7 and P8 had decelerating trends in symptoms during T1, followed by a change in trends in T2. This reflects no effect on symptom reduction during CO-OP sessions.

Interestingly, a floor effect was noted for P2, who had no to minimal depressive and anxiety symptoms from T1 to T2 but experienced a slight increase in anxiety at T4 due to new stressors unrelated to the goals she had previously worked on.

P3 had accelerating depressive and anxiety symptoms in T1, followed by a change in trend to decelerating symptoms in T2. This reflects a positive effect on symptom reduction during CO-OP sessions.

P4 had an accelerating trend in depressive symptoms in T1. T2 was characterized by increased variability, although there was no change in trend and slope. In addition, P4 had decelerating anxiety symptoms in T1, which continued with a similar trend and slope in T2. This reflects no effect on symptom reduction during CO-OP sessions.

P5 had a decelerating trend in depressive symptoms in T1, followed by an increased steepness in the slope in T2. In addition, P5 had accelerating trend in anxiety symptoms in T1, followed by a change in trend in T2. This reflects a positive effect on symptom reduction during CO-OP sessions.

P9 had a decelerating trend in depressive symptoms in T1, which was followed by increased variability in T2. In addition, P9 had a decelerating trend in anxiety symptoms in T2, and a change in trend in T2. This reflects no effect on symptom reduction during CO-OP sessions.

##### 3.6.2.2 Levels

The level of anxiety and depressive symptoms reduced for 62.5% (n=5) of participants between the baseline and at the initiation of the treatment, further indicating a receptiveness to the treatment. When comparing T3 with T4, 50% (n=6) of participants reported maintained or further reduction of the level of depressive symptoms. Moreover, 87.5% (n=7) participants had lower levels of depressive symptoms at 1 month (T4) compared to baseline (T1). A majority 75% (n=6) of participants maintained or had further reduction in levels of anxiety scores between T3 and T4. Moreover, 50% (n=4) participants had lower levels of anxiety symptoms at 1 month (T4) compared to baseline (T1).

Despite most participants not having goals specific to symptom management except for P7 and P9, many noted in their interviews either a reduction in symptoms or increased acceptance of their symptoms. There was a sense of control and personal growth around their symptoms when they were able to use their strategies and participate in desired activities. For example, P1 expressed,

*“But now that I’ve done something, I feel a little less anxious”.*

Similarly, P3 remarked,

*“I think after all the sessions, I’m not so scared of … doing the things that I like to do, in fact I’m like, doing it more often”*.

Additionally, P4 emphasized,

*“I need these strategies, or else I can be pushed back to the place where I first started. That is feeling discouraged. These strategies … change the way I look at things”.*

#### 3.6.3 Confidence

All participants highlighted their active involvement in CO-OP sessions as a positive contributor to their confidence. Having a plan provided structure and encouragement to initiate action towards their goals, while the flexibility to adapt their plans was reassuring for some.

P2 reflected on their initial apprehension, stating,

*“At first, when we started the session I wanted to have someone to follow me [ … ] in case I faint (laughs). But then [after sessions], I say ok what, I take public transport on my own, everything I can do it myself.”*

P3 emphasized how CO-OP bolstered confidence, expressing,

*“It [CO-OP] builds confidence in you la. It gives you confidence, like the things that you’re scared to do, the things that you wish you could do, but never had the chance to do, it’s like they give you the chance to do, then let’s say if this plan is not working, then eh! You got plan B.”*

Although CO-OP enhanced participants’ confidence, some felt the need for additional sessions and guidance to reinforce and refine their strategies. For example, P5 articulated,

*“Because these strategies are new to me so I believe habit takes time, so um no doubt sometimes I feel there’s a bit of a challenge doing that.”*

P1 acknowledged the potential benefits but expressed a need for assistance in developing the strategies, saying,

*“I can probably come up with some ideas but [ … ] I might need help with fixing that to make sure it actually works. But other than that, I think it might do something for my part if I thought about it.”*

Recommendations to bolster confidence were suggested by participants. They included follow-up sessions and involvement of significant others (partners) for support in applying strategies.

Of the eight participants, 75% expressed low confidence in independently transferring GPDC or domain-specific strategies to other situations, advocating for continued therapist support. P9 emphasized the necessity, stating,

*“I need more confidence … that’s why I want [the therapist] to continue with me.”*

P7 advocated for monthly check-in sessions with the therapist to review goals and plans. P1 stressed the importance of professional follow-up, noting that informal support from partners might not be as effective. However, P2 believed follow-up sessions were unnecessary unless new problems arose.

All participants recognized the importance of having a support system, which significantly contributed to intervention efficacy. P1 emphasized this, stating,

*“If I didn’t have the support right, I don’t think I’ll be able to … like reach it, I’ll probably be … like a nervous wreck.”*.

During the intervention phase, several participants involved their partners to aid strategy application or directly as part of their plans. They valued this support, as it helped them execute and refine their plans.

## 4 Discussion

This pilot study explored the feasibility of the CO-OP approach for individuals dealing with mood, anxiety, or adjustment disorders. Overall, the findings suggest that CO-OP holds promise as a feasible intervention. Factors such as demand, efficacy, and acceptability were generally favorable, albeit with some suggested modifications to the intervention format by participants. Despite high fidelity scores indicating adherence to key elements, the therapist encountered challenges during the implementation of CO-OP.

### 4.1 Acceptability

Participants generally embraced the CO-OP approach, favoring it over previously experienced therapy methods. This preference could be attributed to the personalized nature of CO-OP, where participants selected their own goals. They noted that CO-OP offered more flexibility compared to previously experienced approaches, which often felt rigid and difficult to apply in their daily lives. In addition, the active partnership and collaboration also contributed to the acceptability of the approach. The study findings are consistent with existing evidence highlighting the significance of the therapeutic relationship in therapy outcomes (38–42). The therapist’s adept use of skills such as supportive counseling and validation of emotions proved pivotal during sessions, contributing significantly to the acceptability of the CO-OP approach. Moreover, the therapist’s proficiency in discerning the individual’s evolving needs amidst fluctuations in their mental health condition emerges as potentially crucial to the success of CO-OP this population.

However, challenges such as nonattendance persisted. In our study, the eight participants who continued beyond the baseline data collection were all motivated to complete the CO-OP sessions. Reasons given for nonattendance included forgetting appointments or being unwell, suggesting that motivation was less of an issue. This contrasts with other studies that identify motivation as a main contributor to nonattendance in this population (43, 44). Interestingly, other research indicates that adherence to treatmentintensity and frequency has long been a challenge in mental health practice, with studies showing that almost 20% of patients miss their scheduled treatments. This rate is approximately twice as high as that seen in patients with other conditions (45). In our study, participants were motivated to continue CO-OP sessions and complete their goals, although some people did request more time to do this, which was not possible within the timeframe of this study. Thus, the main implications are that more time may be needed to complete the CO-OP sessions for this population and additional administration may also be required to remind participants of their appointments and reschedule missed sessions.

Considering alternative methods of follow-up, such as telerehabilitation, may be beneficial. Telerehabilitation has gained popularity, particularly since COVID-19 (46, 47). Previous research on telerehabilitation for psychiatric interventions has generally shown positive results in terms of effectiveness and feasibility compared to traditional methods (33, 48, 49). However, our study found contrasting results, with low acceptability and demand for telehealth sessions, with participants preferring an in-person interaction. The primary obstacle encountered was concerns about technical difficulties. In future, therapists could explore broader applications of telerehabilitation beyond videoconferencing. This could include utilizing online forums, smartphone apps, text messaging, and emails for delivering mental health services (50, 51).

### 4.2 Efficacy

Through the triangulation of findings from this study, CO-OP yielded clinically significant improvements in both occupational performance and satisfaction ratings on the Canadian Occupational Performance Measure. This echoes findings from previous studies involving stroke and traumatic brain injury populations (26) as well as burn survivors (27), further underscoring CO-OP’s potential efficacy in enhancing occupational performance among individuals with mental health conditions. Notably, most participants reported a reduction in mood and anxiety symptoms, despite not setting goals explicitly related to symptom management. However, the visual analysis of the trends and levels related to the PHQ-9 and GAD-7 suggested that only two participants had symptom reduction during CO-OP sessions. This may indicate that most participants may have experienced heightened symptoms during CO-OP sessions, as the process of problem solving to improve occupational performance could have been challenging, with the successes and setbacks experienced. However, upon reflection, after the completion of the CO-OP sessions, they felt that their symptoms had improved. Furthermore, even for those who did not experience symptom reduction, participants still derived benefits from employing strategies to enhance participation in daily activities and routines. This suggests that there may not be a clear correlation between symptom reduction and occupational performance.

Participants also expressed heightened confidence as a result of the CO-OP sessions. Similar enhancements have been observed in various adolescent and adult populations (28, 33, 49), where participants reported increased confidence in their ability to develop plans and manage daily activities. These improvements were attributed to the autonomy provided by designing their own plans through guided discovery. In our study, framing the intervention as a problem-solving approach may have mitigated participants’ fear of failure, as the CO-OP approach allowed for time and space for experimentation and refinement of plans. Outcome measures associated with confidence could be a useful addition in future studies.

### 4.3 Practicality and implementation challenges

Despite high fidelity, the therapist identified multiple factors that increased the difficulty of implementing CO-OP. These were mainly participants’ symptoms, complex goals, and limited task knowledge. Half of the participants experienced difficulties in learning, generalizing, and transferring GPDC independently. This contrasts with findings in previous studies, where participants across conditions were generally able to do so (21, 29, 33, 52).

As significant portions of certain sessions were spent supporting participants through fluctuations in psychiatric symptoms, the learning of GPDC may have been disrupted. It is possible that participants’ psychiatric symptoms and other co-morbidities (e.g., Autism Spectrum Disorder, Attention Deficit-Hyperactivity Disorder with learning difficulties, Social Communication Disorder) may have been a barrier to learning and generalizing (53). Thus, the therapist identified supportive counseling as vital to prepare participants for engagement in CO-OP.

Moreover, the complexity of goals may have been another factor affecting the learning and application of GPDC and strategies. Similar to a study by Moxham et al. (54), the goals set were mostly social and organizational in nature. It is possible that the number of sessions was insufficient to practice applying GPDC for these complex goals, which are arguably more intricate than the motor-based goals set by children with Developmental Coordination Disorder, for whom the original protocol was designed (12). Additionally, prolonged goal-setting periods for some participants likely diminished the time available for learning and practicing GPDC. To address these challenges, some protocols have been adjusted to include double the number of sessions, as observed in previous studies (29, 55). These studies also incorporated intentional use of prompting questions and discussions to facilitate generalization and transfer beyond the intervention phase.

Furthermore, participants expressed a need for extended therapist support to apply cognitive strategies independently. This aligns with previous findings where participants expressed a desire for a gradual transition towards autonomy (28). Methods to facilitate this transition include booster sessions (29, 56) or a stepped intervention format (57). However, it is crucial to reconsider the overall number of sessions provided, given the prevalent difficulties within this population of non-attendance and their confidence in making life changes. Although participants expressed a desire for more support and ongoing sessions, it remains uncertain whether this would have enhanced occupational performance outcomes or led to problem-solving fatigue. Further investigation is warranted in this regard.

### 4.4 Implications for practice

CO-OP demonstrates promising results as an approach for improving occupational performance among people with mental health needs. When implementing the CO-OP approach in mental health settings, the use of supplementary mental health skills was found to be beneficial in the therapeutic process and in achieving participants’ goals. Emphasizing the development of a strong therapeutic relationship and alliance is essential when delivering CO-OP. This is similar to the emotional support that is a core feature of Occupational Performance Coaching, which is a similar approach where people determine their own goals and use a problem-solving approach (58). Efforts should be made to build trust and create a safe environment during sessions, especially considering that individuals with mood, anxiety, or adjustment disorders may require greater emotional support.

Furthermore, broader adaptations may be beneficial to improve effectiveness in this population. Firstly, there could be more personalization of the intervention format in terms of the number of sessions, intervals between sessions, and involvement of significant others. Secondly, flexibility in delivering CO-OP would be beneficial to strike a balance between problem-solving, addressing participants’ emotional needs, and adherence to appointment schedules.

### 4.5 Study limitations

The study has several limitations, including the limited diversity of participants in terms of gender and the presence of co-morbidities, which may hinder the generalizability of the findings to the broader mental health population. To address these limitations, a more rigorous follow-up study could be conducted, involving a larger and more diverse sample of participants with varying symptom severity and diagnoses. In addition, it will be important for future studies to consider if participant characteristics influence their attitudes and level of adherence to CO-OP. Protocols for future research should also consider the possibility of a high number of missed sessions and the potential differences in telehealth versus in-person delivery. Moreover, involving more than one therapist in the study can help determine if the treatment effect observed in this study can be replicated. Furthermore, to examine the transference of skills and assess long-term retention, future studies could incorporate a longer follow-up period.

## 5 Conclusion

This pilot study highlights the potential of the CO-OP approach as a feasible intervention for people with mood, anxiety, or adjustment disorders. Despite encountering implementation challenges such as nonattendance, and difficulty in learning and applying the problem-solving strategy, the findings underscored several positive aspects of CO-OP, including its acceptability, demand, and limited efficacy. Participants generally embraced CO-OP, favoring its personalized and collaborative nature over previously experienced therapy methods. Notably, the high retention rates observed in this study suggest a strong acceptability of CO-OP.

Practical implications include having an emphasis on building a strong therapeutic relationship using supplementary skills such as supportive counseling and validation of emotions to enhance therapeutic outcomes. Moreover, broader modifications to the CO-OP protocol, such as increased personalization and flexibility, may further enhance its effectiveness in addressing the complex needs of individuals with mental health conditions.

## Data Availability

The datasets presented in this article are not readily available because a data sharing agreement needs to be in place for data sharing. Requests to access the datasets should be directed to SW, suren.wong@singaporetech.edu.sg.
